# Increase in Ribosomal Fidelity Benefits *Salmonella* upon Bile Salt Exposure

**DOI:** 10.3390/genes13020184

**Published:** 2022-01-21

**Authors:** Zhihui Lyu, Jiqiang Ling

**Affiliations:** 1 Department of Cell Biology and Molecular Genetics, The University of Maryland, College Park, MD 20742, USA; 2 Molecular and Cellular Biology, Bilogical Sciences Graduate Program, The University of Maryland, College Park, MD 20742, USA

**Keywords:** translational fidelity, bile salts, pathogen, *Salmonella*

## Abstract

Translational fidelity is maintained by multiple quality control steps in all three domains of life. Increased translational errors (mistranslation) occur due to genetic mutations and external stresses. Severe mistranslation is generally harmful, but moderate levels of mistranslation may be favored under certain conditions. To date, little is known about the link between translational fidelity and host–pathogen interactions. *Salmonella enterica* can survive in the gall bladder during systemic or chronic infections due to bile resistance. Here we show that increased translational fidelity contributes to the fitness of *Salmonella* upon bile salt exposure, and the improved fitness depends on an increased level of intracellular adenosine triphosphate (ATP). Our work thus reveals a previously unknown linkage between translational fidelity and bacterial fitness under bile stress.

## 1. Introduction

The transfer of genetic information from DNA to protein requires optimized levels of fidelity and speed. The rate of amino acid misincorporation during translation in *Escherichia coli* ranges from 1 in 10,000 to 1 in 1000 [1,2]. Although cells have evolved several quality control mechanisms to minimize errors [3,4,5,6], mistranslation still occurs to some extent because of genetic mutations [7,8] and external stresses, such as heat [9] and antibiotics [10]. Under severe mistranslation stress, this can result in the accumulation and aggregation of abnormal proteins that ultimately lead to growth inhibition and cell death [11,12,13]. Therefore, it is commonly thought that mistranslation is detrimental to the cell.

It has been suggested that natural *E. coli* isolates have a wide range of ribosomal fidelity, implying that some degree of mistranslation may be favored under distinct environments [14]. Growing evidence suggests that increased translational errors could be adaptive and beneficial under certain conditions [15,16,17,18]. It is now becoming increasingly clear that the effects of translational errors on fitness depend on both cellular physiology and changes in surrounding conditions [19]. We have recently shown that optimal translational fidelity is pivotal for the adaptation of *Salmonella* to interact with host cells [20]. As an extreme example of a bile-resistant pathogen, *S. enterica* could colonize and survive in the gall bladder during systemic or chronic infections [21,22]. However, the role of translational fidelity in bile salt exposure is unexplored. In the present work, we demonstrate that an increase in ribosomal fidelity enhances the growth of *Salmonella* in the presence of bile salts. We further show that the improved fitness in the high-fidelity mutant depends on an increased level of intracellular ATP.

## 2. Materials and Methods

### 2.1. Bacterial Strains, Plasmids and Growth Conditions

All strains and plasmids used in this study are listed in Table 1. The oligonucleotides used for gene disruption are listed in Table S1. Construction of mutant strains was achieved by lambda Red-mediated recombination [23]. All cultures were grown in Luria broth (LB) medium consisting of 10 g/L tryptone, 5 g/L NaCl, and 5 g/L yeast extract at 37 °C with or without sodium cholate (SC), except for strains containing pKD46, which were grown at 30 °C. Antibiotics were used at the following concentrations: ampicillin, 100 μg/mL; chloramphenicol, 25 μg/mL, and kanamycin, 50 μg/mL. Arabinose was used at 10 mM for induction of Red recombinase by pKD46.

### 2.2. Determination of Mistranslation Rates

The pZS-Ptet-m-TGA-y plasmid was used to determine the translational error rates as described [25]. Plasmid pZS-Ptet-m-y was used as the normalization control, and pZS-Ptet-lacZ was used as a negative control for background subtraction.

### 2.3. Bacterial Dynamic Growth Curve

Overnight cultures were diluted 1:100 in fresh LB with or without sodium cholate and incubated at 37 °C for 16 h with shaking. The effect of sodium cholate on bacterial growth was automatically monitored every 20 min by measuring the optical density at 600 nm (OD_600_) using a BioTek SYNERGY HTX microplate reader (Agilent, Santa Clara, CA, USA).

### 2.4. Liquid Media Competition Assays

Two strains containing yellow fluorescent protein (YFP) or enhanced cyan fluorescent protein (eCFP) were mixed in a 1:1 ratio into 1 mL LB medium in the presence or absence of 20 mg/mL sodium cholate. After 24 h, 1 μL of the mixture was reinoculated into 1 mL fresh medium for another round of 24 h-competition. The relative fitness was analyzed by a BD LSR II flow cytometer (BD, Franklin Lakes, NJ, USA) at a low flow rate. In all, 30,000 gated events were acquired for each sample. Data were further analyzed using the FlowJo software (FlowJo, Ashland, ON, USA).

### 2.5. Measurement of Intracellular ATP

Overnight cultures were diluted 1:100 in fresh LB with or without 20 mg/mL sodium cholate and incubated at 37 °C with agitation for 6 h. Cells were normalized by OD_600_, and 1 mL of culture aliquots were collected by centrifugation (14,000 rpm at 4 °C for 5 min). The pellets were then resuspended in 500 μL of phosphate-buffered saline (PBS) and lysed through boiling at 100 °C for 10 min. The intracellular ATP level was measured using an ATP Determination Kit (Invitrogen, Thermo Fisher Scientific, Waltham, MA, USA) according to the manufacturer’s instruction. Background ATP was subtracted using the readings of blank PBS.

### 2.6. Quantitation and Statistical Analysis

Three independent experiments with at least three biological replicates were performed for each assay. Error bars represent the standard deviation. Statistical differences were analyzed using independent *t*-tests. *p* < 0.05 was considered statistically significant.

## 3. Results

### 3.1. Higher Translational Fidelity Increases Fitness upon Bile Salt Exposure

**Figure 1:**
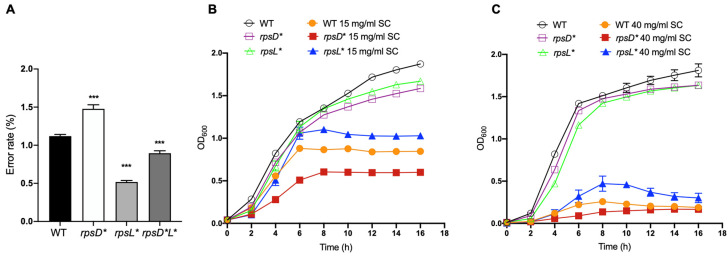
Increased translational fidelity improves growth under bile salt stress. (**A**) UGA readthrough translational errors were measured using dual-fluorescence reporters. Cells harboring the reporters were grown in Luria-Bertani broth (LB) at 37 °C for 16 h. Fluorescence signals were quantified using a plate reader. (**B**,**C**) Growth curve of *Salmonella* strains in the presence of 15 mg/mL or 40 mg/mL sodium cholate. Overnight cultures were diluted 1:100 in fresh LB with or without sodium cholate and incubated at 37 °C for 16 h with shaking. OD_600_ was determined using a microplate reader. The results are the average of at least three biological repeats with error bars representing the standard deviations. The *p*-values are determined using the independent *t*-test. ***, *p*  <  0.001.

To understand the link between translational fidelity and fitness during exposure in bile salts, we separately introduced mutations I199N and K42N into the ribosomal genes *rpsD* (uS4) and *rpsL* (uS12) in the *S. enterica* Typhimurium strain ATCC 14028. We validated the error rate using our dual-fluorescence reporter readthrough assay [25] in the mutant strains. As expected, the *rpsD* I199N (rpsD*) and *rpsL* K42N (rpsL*) strains exhibited increased and decreased rates of UGA readthrough, respectively, when compared with the wild-type (WT) strain (Figure 1A).

To assess the fitness change of altering translational fidelity in *Salmonella* under bile salt conditions, we added various concentrations of sodium cholate into the growth medium. The absorbance at 600 nm (optical density) was recorded at different time points. Without bile salts, the rpsD* and rpsL* mutations slightly decreased growth. However, in the presence of 15 mg/mL or 40 mg/mL sodium cholate, the rpsL* high-fidelity strain exhibited a growth advantage and achieved a higher cell density compared to the WT, whereas the rpsD* strain showed poorer growth than the WT (Figure 1B and Figure S1).

**Figure 2:**
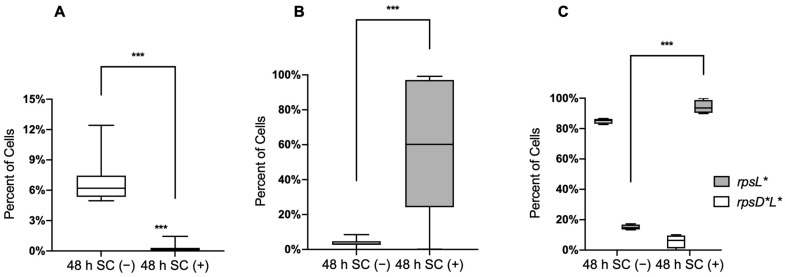
High translational fidelity provides competitive advantage in bile salts. Competition experiments were initiated with a 1:1 mixture of overnight cultures of WT with rpsD*, WT with rpsL*, or rpsL* with rpsD*L* in the presence or absence of 20 mg/mL sodium cholate. Bacteria were grown for a total of 48 h, and bacteria were sorted by flow cytometry. In all, 30,000 gated events were acquired for each sample. (**A**) The error-prone strain rpsD* was outcompeted by the WT strain after 48 h with the addition of sodium cholate. (**B**) The error-restrictive strain rpsL* was outcompeted by the WT without sodium cholate but showed an increased advantage over the WT strain in the presence of sodium cholate. (**C**) Strain rpsD*L* had a higher error rate than rpsL*and was outcompeted by the rpsL* strain in the presence of sodium cholate after 48 h. The data here are representative of results from at least three biological replicates. The *p*-values were determined using the independent *t*-test. ***, *p*  <  0.001.

To further test whether bile salts affect the competition between the mutants and WT in co-cultures, we mixed WT with rpsD* or rpsL* carrying different fluorescence reporters at a 1:1 ratio in the presence and absence of sodium cholate. *Salmonella* cells were grown for a total of 48 h and analyzed using flow cytometry. We found that without sodium cholate, the WT cells outcompeted the rpsD* and rpsL* cells after 48 h, with the fractions of rpsD* and rpsL* cells reduced to 6.8% and 3.6%, respectively (Figure 2). In contrast, in the presence of sodium cholate, the rpsL* strain showed a competitive advantage over the WT (Figure 2A), whereas the rpsD* strain was completely outcompeted by the WT after 48 h (Figure 2B). These results were consistent with the growth curves of individual strains.

The error-prone rpsD* and high-fidelity rpsL* mutations led to opposite effects in *Salmonella* fitness in the presence of bile salts. We next combined the *rpsD* I199N and *rpsL* K42N mutations in a single strain rpsD*L*. The error rate in the rpsD*L* strain was lower than the WT and rpsD* strains but higher than rpsL* (Figure 1A). Interestingly, competition experiments between rpsL* and rpsD*L* revealed opposite trends with and without sodium cholate: the rpsL* strain was outgrown by rpsD*L* without SC but almost completely outcompeted rpsD*L* in the presence of SC (Figure 2C). Collectively, these results demonstrate that increased ribosomal fidelity provides a growth advantage for *Salmonella* under bile salt stress.

### 3.2. Porins, Envelope Stress Response, and Flagella Are Not Major Contributors to Improved Bile Salt Resistance

**Figure 3:**
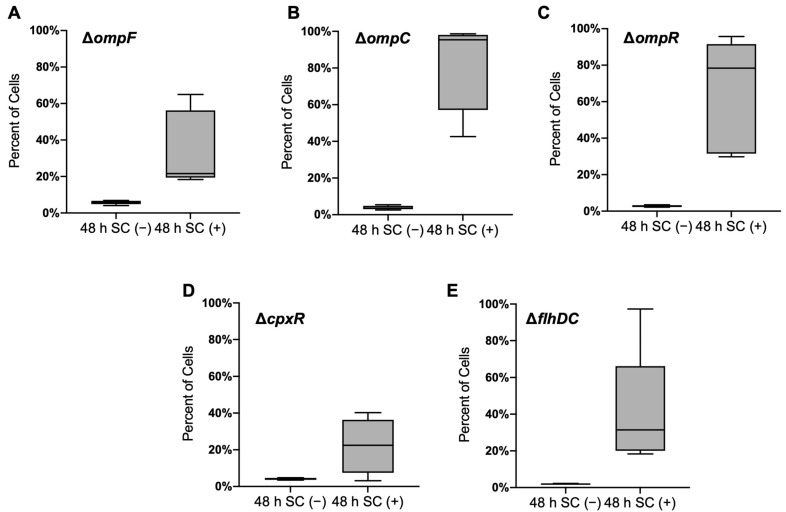
Fractions of rpsL* cells in competition experiments. Overnight cultures of WT Δ*ompF* with rpsL* Δ*ompF* (**A**), WT Δ*ompC* with rpsL* Δ*ompC* (**B**), WT Δ*ompR* with rpsL* Δ*ompR* (**C**), WT Δ*cpxR* with rpsL* Δ*cpxR* (**D**), and WT Δ*flhDC* with rpsL* Δ*flhDC* (**E**) were 1:1 mixed into the 1 mL LB medium in the presence or absence of 20 mg/mL sodium cholate. Bacteria were grown for a total of 48 h and sorted by flow cytometry. The data are representative of results from at least three biological replicates.

In *Salmonella*, the outer membrane porins have been implicated as bile transporters and affect virulence [26]. To test whether those porins are involved in mediating the increased bile salt resistance in the rpsL* strain, we deleted *ompF* and *ompC* genes that encode the most abundant porins from WT and rpsL* *Salmonella*. As shown in Figure 3A,B, deleting *ompF* or *ompC* did not affect the increase of rpsL* fraction in the presence of sodium cholate. Porin expression is also regulated by the two-component system EnvZ-OmpR in response to changes in osmolality and pH [27,28,29]; we found that deleting *ompR* did not alter fitness gain by the rpsL* mutation in the presence of sodium cholate either (Figure 3C).

It has been suggested that a bacterial envelope provides a barrier that restricts the entry of bile salts [30,31,32], and bile salts could disrupt the integrity of both the outer and inner membranes leading to envelope stress [33,34]. Translational fidelity may affect protein misfolding and the CpxA-CpxR envelope stress response. Nevertheless, we found that the sodium cholate still enriched rpsL* cells when *cpxR* was deleted from the WT and rpsL* strains (Figure 3D), suggesting that the envelope stress response is not fully responsible for the increased resistance to bile resistance in high-fidelity cells.

Our previous study demonstrates that the assembly of flagella consumes the proton motive force and decreases the efflux pump activity, leading to antibiotic sensitivity [35]. The rpsL* mutation decreases the expression of flagellar genes and swimming motility [20]. Such trade-off between motility and efflux prompted us to investigate whether the master flagellar regulator *flhDC* was involved in bile resistance. The results revealed that the deletion of *flhDC* did not appear to decrease the fitness of rpsL* cells in the presence of sodium cholate (Figure 3E), suggesting that another pathway may lead to bile resistance in high-fidelity cells.

### 3.3. Improved Fitness in High-Fidelity Strain Depend on Increased Intracellular ATP

**Figure 4:**
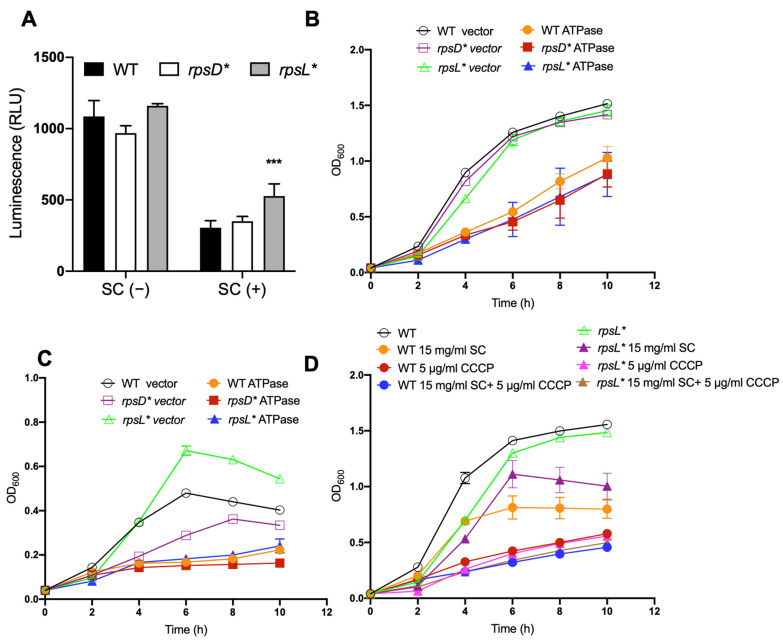
Improved fitness in error-restrictive strain depends on increased ATP levels. (**A**) Determination of intracellular ATP levels. Overnight cultures were diluted 1:100 in fresh LB with or without 20 mg/mL sodium cholate and incubated at 37 °C with agitation for 6 h. The cellular ATP level (indicated by the luminescence signal) decreased significantly in all three strains after exposure to bile salts, and the rpsL* strain maintained the highest level of ATP. (**B**,**C**) Growth curve of *Salmonella* strains harboring plasmid of IPTG-inducible ATPase. Overnight cultures were diluted 1:100 in fresh LB supplemented without (**B**) or with (**C**) sodium cholate and incubated at 37 °C for 10 h with shaking. In total, 1 mM IPTG was added to induce the expression of ATPase for ATP hydrolysis. (**D**) Growth curve of *Salmonella* strains with and without CCCP to inhibit ATP synthesis. The data are representative of results from at least three biological replicates. The *p*-values were determined using the independent *t*-test. ***, *p*  <  0.001.

Translational errors affect protein misfolding and aggregation [13,36]. Interestingly, it has also been well established that bile salts cause misfolding and the denaturation of proteins [34,37]. Misfolded proteins harm bacterial cells and rely on ATP-dependent chaperones and proteases for refolding or degradation [38]. We thus examined the ATP levels in WT, rpsD*, and rpsL* cells grown in the presence and absence of sodium cholate. We observed that the cellular ATP level decreased substantially in all three strains after exposure to bile salts, and the rpsL* cells maintained the highest level of ATP among the three strains (Figure 4A). It is likely that the high-fidelity rpsL* cells generate less misfolded proteins in the presence of sodium cholate, therefore consuming less ATP during protein quality control.

To further test whether the cellular ATP level contributes to improved fitness of high-fidelity cells after exposure to bile salts, we used a plasmid encoding the soluble subunit of ATPase to cause IPTG-inducible ATP depletion [24]. In the absence of sodium cholate (Figure 4B and Figure S2), the depletion of ATP caused the retarded growth of all three strains compared to the vector control. In the presence of sodium cholate (Figure 4C), rpsL* cells carrying the vector control exhibited improved growth compared to the WT, but this growth advantage was abolished upon induction of the ATPase. In line with this, we used a protonophore CCCP, which uncouples the proton motive force across the cellular membrane and reduces ATP synthesis to treat the WT and rpsL* strains. As shown in Figure 4D, after CCCP treatment, the rpsL* strain no longer exhibited improved growth when exposed to bile salts. Taken together, these results suggest that a higher cellular ATP level in the rpsL* strain contribute to its improved fitness after exposure to bile salts.

## 4. Discussion

Bile is a fluid synthesized by hepatocytes with antibacterial capacity due to the presence of bile salts [30,37]. Bile plays an essential role in the digestion and absorption of fat and soluble vitamins in the intestine [39]. It has the ability to dissolve membrane lipids and cause the denaturation of proteins [34,37]. *Salmonella* is a bacterial pathogen that causes tens of millions of gastrointestinal infections in humans worldwide each year [40,41,42]. During its systemic or chronic infections, *Salmonella* is exposed to bile salts [21,22], suggesting that they might have bile resistance mechanisms to facilitate their colonization in this environment. The establishment of a novel niche for replication in gallbladder epithelial cells may help *Salmonella* escape from the extremely high concentrations of bile salts present in the gall bladder lumen [43]. Biofilm formation on the surface of cholesterol gallstones may also protect *Salmonella* from the bactericidal activities of bile salts [44,45]. However, planktonic *Salmonella* cells are also found in the gall bladder lumen, implying that some unknown mechanisms are employed to permit their survival and replication. More recent studies show that some degree of translational errors may improve fitness by protecting cells from oxidative damage and antibiotics [18,19,46,47], which prompted us to investigate the role of translational fidelity in bile salt exposure. In the present work, we demonstrate that the error-prone strain rpsD* and error-restrictive strain rpsL* have decreased and increased fitness upon bile salt exposure, respectively, when compared to the WT (Figure 1 and Figure 2).

Genetic and biochemical studies have revealed a number of bile resistance factors in enteric bacteria, including porins [48], efflux pump systems [49], lipopolysaccharide [31], multiple antibiotic resistance (*mar*) gene [50], and *phoPQ* [51]. In this study, we show that the major outer membrane proteins OmpF and OmpC are not mainly responsible for the increased bile salt resistance in the rpsL* strain (Figure 3). Instead, we show that high translational fidelity lowers the amount of ATP consumption in the presence of bile salts (Figure 4A), likely through restricting protein misfolding. The resulting higher ATP level in the high-fidelity cells, in turn, benefits growth under bile stress, as suggested by our ATP depletion results (Figure 4C,D). Our work, therefore, reveals a previously unknown impact of translational fidelity on bacterial fitness under stress conditions, with implications in *Salmonella*–host interactions.

## Figures and Tables

**Table 1:** Strains and Plasmids used in this study.

Strains	Source or Reference	Genotype/Features
*S. typhimurium* 14,028 s (WT)	ATCC	N/A
rpsD*	Lab collection	*rpsD* I199N
rpsL*	Lab collection	*rpsL* K42N
rpsD*L*	Lab collection	*rpsD* I199N and *rpsL* K42N
Δ*ompF:kan*	This study	Region 1,048,143 to 1,049,228 (Δ2–363 aa)
Δ*ompC:cat*	This study	Region 2,416,999 to 2,418,129 (Δ2–378 aa)
Δ*ompR:cat*	This study	Region 3,673,507 to 3,674,220 (Δ2–239 aa)
Δ*cpxR:cat*	This study	Region 4,283,333 to 4,284,025 (Δ2–232 aa)
Δ*flhDC:cat*	Lab collection	Region 2,032,540 to 2,033,471 (Δ*flhD* 1–117 aa Δ*flhC* 1–193 aa)
**Plasmids**	**Source or Reference**	**Genotype/Features**
pKD46	Lab collection	ampR
pKD3	Lab collection	ampR and camR
pKD4	Lab collection	ampR and kanR
pZS-Ptet-m-y	Lab collection	ampR
pZS-Ptet-m-TGA-y	Lab collection	ampR
pZS-Ptet-lacZ	Lab collection	ampR
pZS-Ptet-YFP	Lab collection	ampR
pZS-Ptet-eCFP	Lab collection	ampR
pUHE-ATPase	[24]	ampR
pVector	[24]	ampR

* Denote specific strains with mutations in rpsD (I199N) and rpsL (K42N) genes.

## Data Availability

Data supporting the findings are within this paper and supplemental files.

## Supplementary Materials

The following are available online at https://www.mdpi.com/article/10.3390/genes13020184/s1, Figure S1: Growth curves of *Salmonella* strains in the presence of sodium cholate. Figure S2: Growth curves of *Salmonella* strains. Table S1: Oligonucleotides used in this study.
