# Supplementary material for: Increase in Ribosomal Fidelity Benefits Salmonella upon Bile Salt Exposure
Source: Genes (Basel). 2022 Jan 21;13(2):184. doi: 10.3390/genes13020184 (PMC8872077; doi:10.3390/genes13020184)
Supplement: Supplementary file 1 [file genes-13-00184-s001.zip › genes-1560501-supplementary.pdf]

Supplementary Material

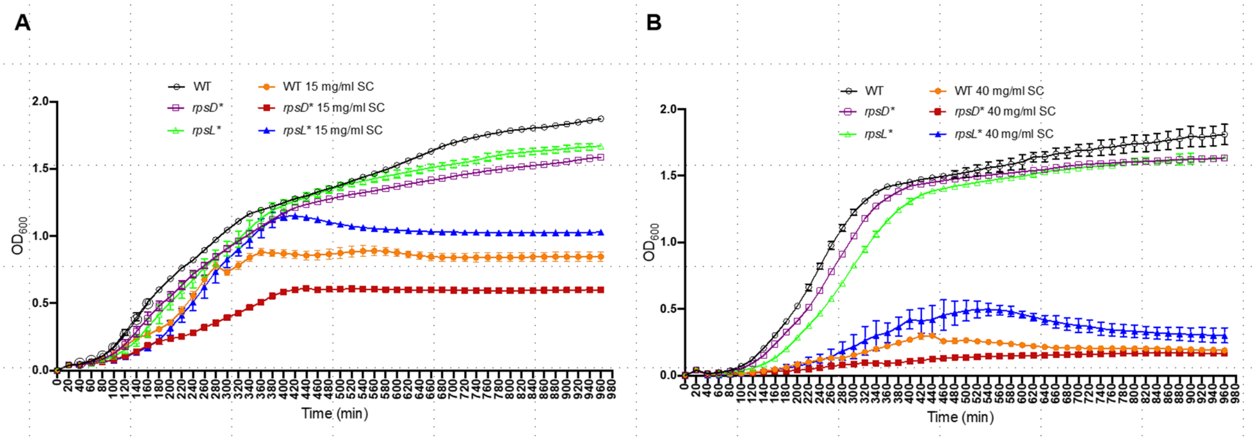

Figure S1. Growth curves of *Salmonella* strains in the presence of sodium cholate. (**A**) and (**B**) are from the same data as Figures 1**B** and 1**C**, respectively, except that more time points are shown.

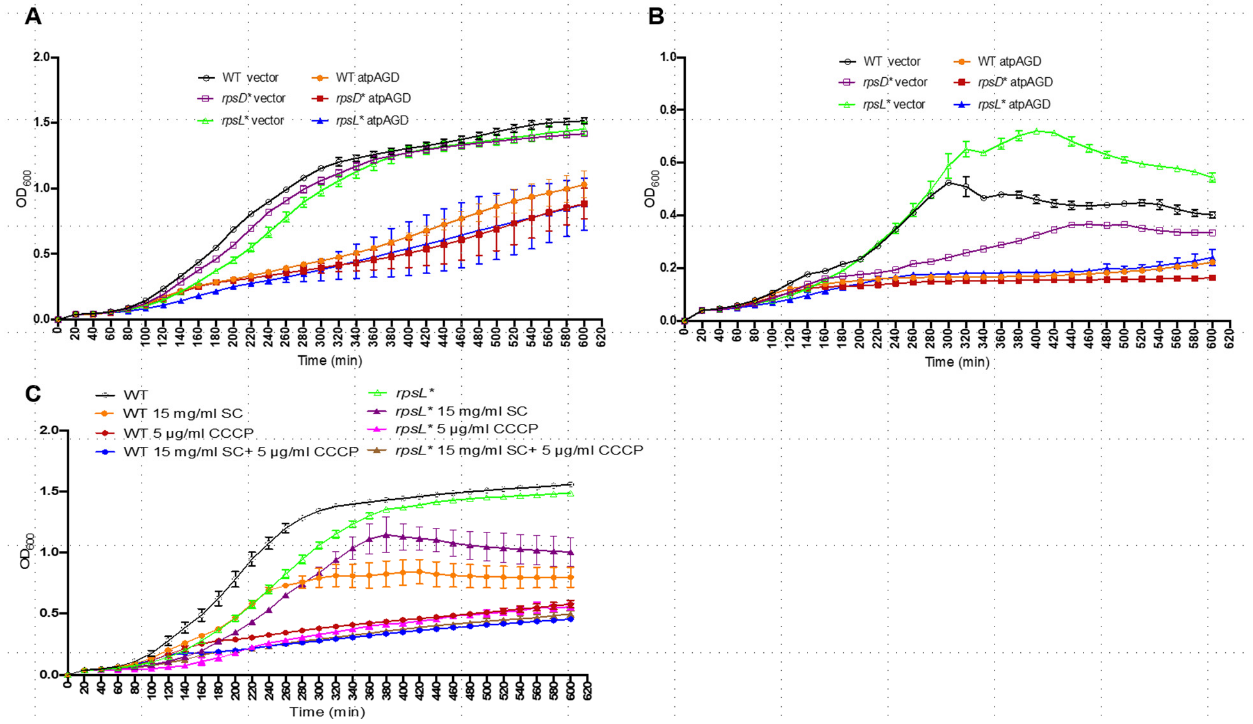

Figure S2. Growth curves of *Salmonella* strains. (**A–C**) are from the same data as Figure 4**B–D**, except that more time points are shown.

Table S1. Oligonucleotides used in this study.

Oligonucleotides Name	Oligonucleotides sequence (5′-3′)	Note
ompF-P1	gcagtggcaggtgtcatataaaaaaaccaatgagggtaataaataatgtcttgagcgattgtgtagg c	For *ompF* deletion
ompF-P2	gaccgaagtcctgtttttgaggcataaaacaaaggggtctgctgatcacatatgaatatcctccttag	
ompF-F	taatgatgaagcgcaaaatcct	For verifiying *ompF* deletion
ompF-R	atcagaactggtaagtaatacc	
ompF-U	caaatcaaaacgataactcagg	
ompF-D	ttatgttctcaaacatgacgag	

ompC-P1	gtggcataaaaaagcaataaaggcatataacagagggttaataacatgATGGGAATTAGCCATGGTCC	For *ompC* deletion
ompC-P2	catgaaaaaagggcccgcaggccctttagcaacatcttttgctgattaTGTAGGCTGGAGCTGCTTCG	
ompC-F	ttaaagtactgtccctcctgg	For verifiying *ompC* deletion
ompC-R	agaactggtaaaccagaccc	
ompC-U	ctatgtagataactgtaacatct	
ompC-D	aaaaagtcattttcatcgctgtt	
ompR-P1	agaatacacacttacatttgttgcgaacctttgggagtacagacaatgATGGGAATTAGCCATGGTCC	For *ompR* deletion
ompR-P2	acagcgtgcgggcaaatgaacttcgcggtgagaagcgcattcgcctcaTGTAGGCTGGAGCTGCTTCG	
ompR-F	gattctggtggttgatgacg	For verifiying *ompR* deletion
ompR-R	agaaccgtccggtacaaaga	
ompR-U	cacacggggtataacgtgat	
ompR-D	ttggtgtagagagagatccc	
cpxR-P1	agcgacgcctgatgacgtaatttctgcctcggaggtacgtaaacaatgATGGGAATTAGCCATGGTCC	For *cpxR* deletion
cpxR-P2	gcgtcaaccagaagatggcgaagatgcgcgcggttaaacttcctatcaTGTAGGCTGGAGCTGCTTCG	
cpxR-F	aatcctgttagttgatgatgac	For verifiying *cpxR* deletion
cpxR-R	catgaagcggaaaccatcag	
cpxR-U	aactgaatgccagcgttgag	
cpxR-D	cacttatcgatcgcgcggaa	
